# Coping strategies and perceived barriers of women hospitality workplace employees to sexual harassment in Bahir Dar city, Ethiopia: a grounded theory approach

**DOI:** 10.1186/s40359-021-00648-w

**Published:** 2021-09-16

**Authors:** Mulugeta Dile Worke, Zewdie Birhanu Koricha, Gurmesa Tura Debelew

**Affiliations:** 1 Department of Midwifery, College of Health Sciences, Debre Tabor University, Debre Tabor, Ethiopia; 2 Department of Health, Behavior, and Society, Faculty of Public Health, Jimma University, Jimma, Ethiopia; 3 Department of Population and Family Health, Faculty of Public Health, Jimma University, Jimma, Ethiopia

**Keywords:** Unwanted sexual attention, Normalisation, Engagement, Help-seeking, Detachment

## Abstract

**Background:**

Coping depicts how people detect, appraise, deal with, and learn from stressful encounters. Applying preferred coping strategies in various situations makes the issue a persistent agenda in hospitality workplaces, where women are unduly victims of sexual harassment. Thus, this study aimed to develop a context specific and data-driven coping strategy framework and barriers to coping strategy mechanisms for sexual harassment victimisation against women working in hospitality workplaces.

**Methods:**

A qualitative, grounded theory approach was used. Data were collected from female employees, managers, cashiers, and customers. Semi-structured focus-group discussions and in-depth interview guides were employed. A constant comparative approach was used to describe the meanings and summarise the data. Data were coded, categorised, and networks were visualised using the ATLAS ti version 8.4.24 software package.

**Results:**

In this study, six focus group discussions, ten in-depth interviews, and thirteen key informant interviews were conducted. The provided context specific coping strategic framework consists of four strictly interconnected dimensions with corresponding barriers practised by female hospitality employees. These were normalisation, engagement, help-seeking, and detachment. The normalisation dimension encompasses silence, acceptance, denial, refusal, grief, and tolerance. Confrontation, negotiation, retaliation/threatening, and discrimination of the perpetrators were included in the engagement dimension. Elements such as discussing with friends, complaining with supervisors, consulting professionals, and accusing perpetrators were in the help-seeking dimension. Lastly, job-hopping, job withdrawal, work withdrawal, and distancing were in the detachment dimension. Some barriers deterred all dimensions, some factors facilitated normalisation, and some adverse outcomes ended the engagement dimension.

**Conclusion:**

Our study demonstrated that the coping capacities of sexual harassment among female hospitality employees have been apparent, providing space for stakeholders to intervene. Our new coping strategy framework can serve as a valuable guide for designing context-specific interventions. These interventions could help women and stakeholders prevent sexual harassment, decrease barriers, and alleviate these effects.

## Background

Countries, and unions worldwide, such as the United States of America [1], India [2], African Union [3], Philippines [4], and European Union [5], considered occurrences such as any unwanted verbal, non-verbal, or physical behaviour with the intent or effect of violating the person’s sexual dignity, in particular, the occurrence of an intimidating, hostile, degrading, humiliating/offensive environment is workplace sexual harassment (WSH). As we do in the current paper, it was also widely defined as sexual assault, rape attempts, and rape [6]. In short, WSH is a condition of an unwelcome sexual advance, request for sexual favours, or hostile verbal or physical action that harms one’s job performance or employment [7, 8]. Currently, the literature provides convincing evidence for the persistence and pervasiveness of WSH [8–11]. Evidence also indicates that WSH against women could have overwhelming effects on their safety, health, well-being, and, ultimately, their work [8, 12]. The consequences of WSH are summarised as emotional, psychological, professional, and health-related [8, 13], which incur costs globally, and significant costs in low and middle-income countries [14]. Decrements in risk among vulnerable individuals require well-established social assets, including social networks and tailored reproductive health knowledge [15].

One of the embodiments of WSH is the hospitality workplace [10–12]. Given the increasing number of hospitality industries and more women’s enrolment than men’s, there is a growing concern that WSH may increase the prevalence and severity of its impacts [12]. Though it could affect women everywhere, those working in the hospitality industry are the most vulnerable, unorganised (female, young, and minorities) with income insecurity which emphasises their dependency on supervisors, managers, and customers [16]. However, due to differences in perception, experience, and coping strategies based on gender, context, and ideology, it is still a debatable and unsettled issue worldwide, particularly in low and middle-income countries [8, 17].

The WSH could be caused by the structure, manager, beliefs, and norms in the hospitality workplace [16]. The structural causes are either related to the structure of the hospitality workplace or its employment. On the other hand, managerial causes could be either from seeing violent behaviour as an acceptable managerial practice or perceiving WSH due to failed management and weak leadership. Furthermore, universal norms and beliefs of the hospitality workplaces include the belief in aggressive behaviours as part of the job in the hospitality industry; the belief that staff should obey guests’ wishes (‘the customer is always right’ norm), and the belief that the manners of customers are acceptable and should be tolerated [16]. These causes, brokers’ involvement in the perpetrators’ group, and employee engagement in transactional sex practice made the issue worse than ever. Consequently, WSH harms organisations and individuals.

Although all individuals (i.e., employees, supervisors, customers, and witnesses) could be affected, the effect is worse among victims (employees) [18]. The effects include feelings ranging from embarrassment and anger to disgust, adverse feelings about work, and feeling cheap. It also affects an individual’s employment (regarding security and promotions) and interferes with an individual’s work performance, interpersonal well-being, and interpersonal relations due to significant psychological upset [19]. Studies have revealed a link between WSH and women’s psychology, such as post-traumatic stress symptoms and other mental health aspects [20–25]. Victims of sexual harassment face both immediate and chronic psychological consequences [26, 27]. The immediate psychological effects are shock, denial, fear, confusion, anxiety, withdrawal, shame/guilt, nervousness, distrust of others, and disorder symptoms such as emotional detachment, sleep disturbances, flashbacks, and mental replay of assault [28, 29]. The chronic psychological consequences are depression, generalised anxiety, suicide (attempted or completed), post-traumatic stress disorder symptoms, diminished interest/avoidance of sex, and low self-esteem/self-blame. Certain aspects of WSH’s organisational climate and appraisal were also significant predictors of psychological symptoms [30]. This creates an offensive work environment, especially for women. At the firm level, the individual’s consequences for employees led to higher absenteeism, increased staff turnover, reduced productivity, poor industrial relations, a growing number of complaints and litigation, and poor public relations [31]. From a sector perspective, the high rates of violence and harassment create a sexualised and risky image for the working environment and deter potential workers who cannot tolerate these behaviours [32].

Given the ubiquity, multidirectional cause, and multiple and all-rounded effects of sexual harassment, the ways victims respond/cope were different [8]. The difference in responding/coping starts with the difference in definitions. Some define coping as attempts to neutralise stress or as an action that protects people from being psychologically or emotionally harmed [33]. Others characterise coping as a critical component of adaptation and survival that describes how humans recognise, assess, cope with, and learn from stressful situations [34]. Others also define coping strategies as psychological patterns that people adopt to manage their thoughts, feelings, and behaviours as they go through different stages of illness and therapy [35].

Consequently, coping strategies are numerous and varied as the stressors that precede them [36]. Accepting the situation or one’s role in it, active coping aimed at removing the stressor or oneself from the stressor, anticipatory coping aimed at an expected but uncontrollable event, avoiding/escaping the stressor or associated feelings of distress, denying the problem or feelings, disengaging mentally or behaviorally (giving up), and distancing/detaching from the situation or one’s role in it are just a few examples [37, 38]. It also includes interpreting the stressor as a positive or growth-oriented experience, seeking social support, controlling one’s emotions or waiting for the right time to act, using substances to dull feelings, suppressing competing activities until the problem subsides, turning to religion, using humour, and venting emotions [36–38]. Coping strategies were also grouped based on function (problem-focused, emotion-focused, dysfunctional coping) [39], the direction of response concerning the threat (approach coping and avoidance coping) [40], orientation (task-oriented, emotion-oriented, and avoidance-oriented) [41, 42], and comprehensively as voluntary coping vs involuntary coping, engagement vs disengagement coping, and primary control coping vs secondary control coping strategies [43]. Recently, as coping models/strategies have become more elaborated, coping research is continuously moving toward the view of coping as a multifaceted process [44]. The choice of specific coping strategies used in response to WSH varied significantly depending on occupational status, gender, climate, harassment severity, and power differential. These strategies could be determined by the personal (occupational status of the target within the organisation, race, and gender), environmental (the climate of the organisation in which the harassment occurred, the severity of the harassment incident, and the difference in power between the perpetrator and the target), and cognitive (cognition, arousal, and emotional reactions) factors [45, 46]. There are also debates about whether coping is a single act or process [8]. Similarly, studies consider reporting as a last resort [8, 17, 47]. However, though the responses differ based on the severity of the harassment, victim’s ideology, victims’ perception, the work environment, and the type of coping strategy, of all coping strategies, help-seeking has been buffered the effects of workplace sexual harassment on health and work-related outcomes [48, 49], thereby diminishing adverse consequences among victims.

In Ethiopia, despite workplace sexual harassment’s prohibition by the criminal code proclamation number 414/2004 prohibiting SH and prescription of simple imprisonment [26], it considered a prohibited act of workplace under proclamation number 1156/2019 [50] and contributed 4.1% of the total gross domestic product, and 8.4% of the total employment, WSH in the hospitality workplace was a hidden problem until quite recently. The magnitude of workplace violence was reported only by a few studies among commercial sex workers [51, 52], health care providers [53–55], restaurant workers [56], university students [57], and female faculty and staff [58] in limited areas of Ethiopia, and none of these studies recognised employees’ coping strategies. They also centred on communities that received relatively more attention. However, most interventions focus on reducing reproductive health problems such as sexually transmitted infections (STIs), including human immunodeficiency virus/acquired immune deficiency syndrome (HIV/AIDS), unsafe abortion, and unwanted pregnancy, but do not focus on WSH. However, these issues are essential for designing effective WSH prevention programs among women working in hospitality workplaces.

Therefore, this study aimed to understand coping strategies, barriers, and facilitators as a basis for informing the development of data-driven and context-specific coping strategic framework dimensions, which could provide potential WSH prevention pathways among women who have been working in the hospitality workplaces of the urban city administration, in northwestern Ethiopia.

## Methods

### Study setting

This qualitative grounded theory approach was conducted in Bahir Dar city, the Amhara national regional state’s capital, located 565 km away from Ethiopia’s capital, Addis Ababa, in the North West. The city is located at 11°36′ latitude and 37°37′E longitude at 1820 m above sea level. It has 9 Sub-cities and 17 Kebeles (the smallest administrative unit). The population of the city is 356,757 (296, 532 urban and 60,225 rural). It is a tourist destination city in the state. Rural to urban migration increases in the city, making the city a prominent tourist destination that offers many recreations and hospitality-driven industries. Most hospitality workplaces are situated in the town, mainly because the city’s recreational centres are favourable for enjoyment. The estimated number of women working across these different facilities ranged from 12 to 40. All hospitality workplaces were chosen as the settings for this study. The selected hospitality workplace conditions were not known at the beginning. These hospitality workplaces were screened based on their compliance conditions imposed by the Ministry of labour service authority and hospitality workplace management’s willingness to participate in this study.

### Design

This qualitative study was conducted within the framework of Strauss and Corbin’s grounded theory. This method recognises the existence of multiple socially constructed realities. It aims to elaborate interpretations that can clarify the behaviours of the study participants and describe the processes. Grounded theory is suitable for exploring areas or fields that have not been examined previously or that need to be explored in greater depth, even from a new perspective [59]. The “Consolidated criteria for reporting qualitative studies (COREQ)” 32-item checklist was employed for reporting [60].

### Population and sampling

Data were collected using focus group discussions (FGD), in-depth interviews (IDI), and key informant interviews (KII). Ten IDIs and six FGDs were conducted with female hospitality workplace workers. These workers had at least six months of working experience in the hospitality industry. The participants were currently working in hospitality workplaces in the study area. Women working in the hospitality sector were identified and interviewed. A non-governmental organisation’s community workers living in the city where the study participants live help reach them. Thirty-five female hospitality workplace’s workers participated in the FGDs. Five participants were in two FGDs, seven were in one FGD, and six were in the other three. Thirteen KIIs (five managers, four cashiers, and four customers) were conducted. Four hospitality workplaces were visited to conduct interviews with the supervisors. Initially, the managers/supervisors’ willingness to be questioned was rejected. Nevertheless, permission was granted with personal networks, effective management, and convincing that the researcher was a PhD student with ethics approval from the University. Hospitality workplace managers welcomed an interview after knowing the identity of the researcher.

A purposeful sampling method was used in the initial interviews. According to the emerging codes and categories, the required data were collected using theoretical sampling. Participant selection, data collection, and data analysis were continued until theoretical saturation was reached. A detailed and accurate description of the experience was obtained.

### Participant’s characteristics

Fifty-eight respondents participated in the FGDs, IDIs, and KIIs. The participant women’s ages ranged from 18 to 30 years. Managers, cashiers, and customers who worked as merchants, tour guides, and drivers were among the key informants. The participants’ characteristics have been published elsewhere [61].

### Ethics

The ethical and safety requirements for researching sensitive topics from the world Health organisation have been observed [62]. Debre Tabor University’s Institutional Review Committee (IRC) approved this ethical issue (Ref No-RP/366/10). All participants received information sheets, written consent was obtained from each study participant before their participation, and written consent was approved to record the conversation and transcription. To ensure data privacy, anonymity, and confidentiality, the research team informed each respondent that their identities and the evidence they would provide would be kept confidential. The participants were also told that only the researchers who were actively involved with this study would access it. They were informed that there were no ‘right’ or ‘wrong’ responses. Information was also given concerning their right to terminate participation or withdraw their data before data analysis. This explanation contributed to the creation of a safe environment for women to discuss personal work experiences. After the data was obtained, the field notes, transcripts, audio recordings, and subsequent publications were de-identified to ensure confidentiality. Instead of using their identities, the researchers used generic terms such as “study participants’ and “female workers’ instead of their names in this article. Privacy and confidentiality were ensured by conducting FGDs and IDIs away from their work. Furthermore, the research team contacted psychosocial and medical treatments for women who had revealed severe issues such as rape or difficulties in dealing with sexual harassment.

### Data collection and procedure

This study employed qualitative ground theory. The bias that may appear by using a single method was reduced by applying multiple data collection methods [62, 63]. Similarly, the limitations of using any single method were avoided by using multiple data collection methods. FGDs were first directed to place issues in a group context where comfortable women could share their experiences and ideas. Following the FGDs, the IDIs were conducted with separate women workers. As a result, they were able to squeeze out more in-depth about their workplace experiences. Both IDIs and FGDs were performed to realise the individual and group perspectives of their sexual harassment coping strategies, perceived barriers, and facilitators of response to sexual harassment during work. In contrast, KIIs were undertaken to understand better workplace relations and power dynamics related to sexual harassment response at work from hospitality workplace supervisors/managers, cashiers, and customers.

From January to August 2019 was the data collection period. The expanded data collection period was primarily due to difficulties accessing women due to their very long working hours. Similarly, it was due to problems in accessing KIIs due to competing priorities for their time.

Interview guides were intended to elicit discussion with study participants. The guides developed for IDIs and FGDs were similar, but a distinct interview guide was developed for KIIs. The issues covered were employees, managers, co-workers, and customers’ responses to WSH. Similarly, it included barriers and facilitators of employees to respond to those experiences. Furthermore, the ideas of what could be done to prevent or better respond/cope with WSH were included. All guides were pre-tested with five people who had similar demographic profiles. The pre-test was planned to assure their suitability to improve the guidelines and interview techniques for the local setting. All discussions were conducted in Amharic, the local language. In-depth interviews and focus group discussions with women were held in a convenient location for the study participants. The In-depth interviews and focus group discussions were held in a hotel where women workers felt safe and protected. The study participants sought to go away from their work environment to have open discussions about their experience of coping with WSH. The researchers also wished to avoid the formality of the hospitality workplace environment. Therefore, the researchers ran the FGDs to find women who could perform the treatment safely and competently. The researchers also attempted to make the location in a common area where all participants could use public transportation. Interviews with key informants were conducted in a private room provided freely by the hospitality workplace managers/supervisors.

Appointments were made over the phone for each participant. Four researchers: the principal investigator, and three female qualitative study experts conducted the FGDs and the IDIs (two for each): one facilitated the discussions. The other assisted with the logistics of getting together the women and taking notes as required. All interviews were audio-recorded with consent from participants. Each IDI, KII and FGD lasted roughly 60–105 min, with an average interview time of 80 min. Participants were offered tea, coffee, water, and a soft drink bottle and covered their transportation costs. Both FGDs and IDIs were conducted during the daytime and in the evening (until 8:00 PM).

### Data analysis

All recorded interviews and FGDs were transcribed, approved by the researchers, and then analysed. Before coding, the texts were cross-checked with audio files for accuracy and consistency. A research assistant who was a university graduate with experience in conducting qualitative research and the principal investigator prepared the copies. Data were analysed by following a grounded theory approach suggested by Corbin and Strauss [64]. Data collection and analysis were performed simultaneously. The data from each interview was analysed before the following interview was started. Unanswered questions from the initial interview were emphasised more in the following interview. Accordingly, the first interviews directed the subsequent interviews. Open coding, axial coding, and selective coding were used in the current study.

First, open coding was performed by line-by-line coding after each interview. Then, the data were split to compare the incidents and examine the similarities and differences in the data’s beginning patterns. Codes were immediately built into the English language to facilitate the involvement of all authors. The first two transcripts were coded autonomously by two people, the first author (MD) and the research assistant fluent in Amharic and English. Until consensus was reached on the codebook, several meetings were directed with all the authors. We followed the constant comparative method for data collection and analysis [64].

A codebook was built along with the interview sessions and regular discussions with the authors. At this stage, a total of 403 initial codes were salvaged inductively. Similar codes were merged, and meanings were assigned to the data, resulting in 46 subcategories grouped into four categories based on their commonalities.

Strauss and Corbin’s paradigm scheme was used for axial coding to identify the core categories, theoretical data saturation, constant comparative analysis, constant sensitivity, and memoing [64], which provides general building blocks to formulate a specific hypothesis. These identified blocks were normalisation, engagement, help-seeking, and detachment. At this stage, relationships were identified between the categories, and the analysis was refined.

Finally, selective coding was commenced, referring to integration and refining the theory using a storyline as a tool. A core phenomenon was carefully chosen through several team meetings. It must be central and connected to all the others, frequently appear in the data, be logical and reliable, have explanatory power, and explain variations [64]. All authors discussed the relation of each category with each other until the storyline was well-defined. Finally, we refined the scheme and validated it to maintain internal consistency and logic. To organise the data, we used ATLAS. ti 8.4 computer software.

### Rigour

For dependability and authenticity, Guba and Lincoln’s criteria were followed [65]. We triangulated the researchers, data collection techniques, and data. The transcriptions were sent by e-mail to the people participating in the interview and focus group discussion on confirming the contents. The analysis was supported by the memorandums written during the entire analytical process. The researchers applied reflexivity during the analysis to avoid biases related to their experiences of the phenomenon under study [66].

## Results

### Coping strategic dimensions and barriers

**Fig. 1:**
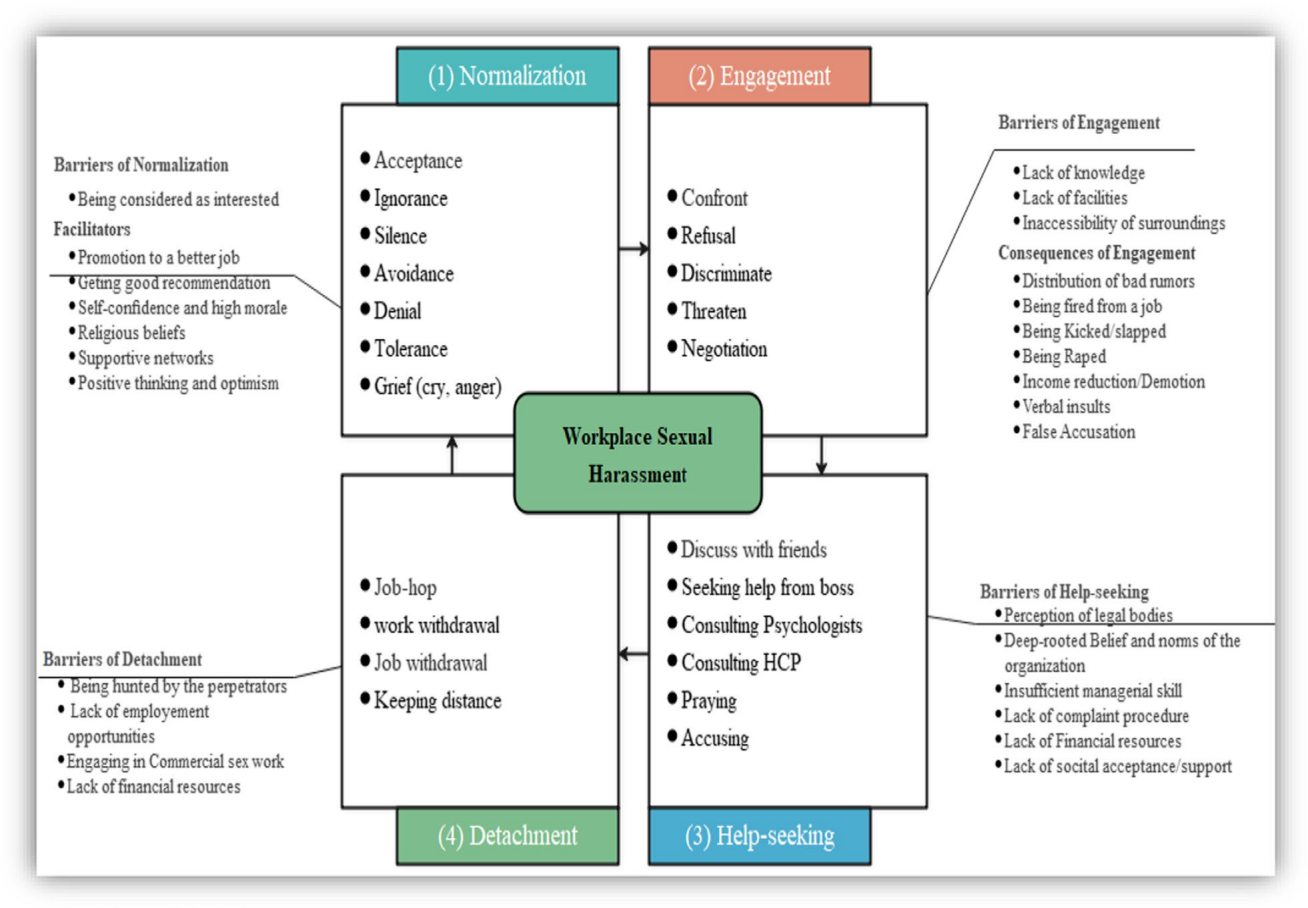
Strategic coping dimensions and sexual harassment barriers among women working in hospitality workplaces, Bahir Dar, Northwest Ethiopia

The findings are organised in a framework named the workplace sexual harassment coping strategic framework. This strategic framework was developed after the coding and clustering codes for women’s sexual harassment victimisation responses. The identified coping dimensions were (1) normalisation, (2) engagement, (3) help-seeking, and (4) detachment. Normalisation is the dimension through which ideas and behaviours that may fall outside social norms are regarded as “normal.” Engagement is oriented toward addressing sexual harassment. These might include confronting the source or the perpetrators of sexual harassment (e.g., challenging the perpetrators about sexist behaviour, including accusing his unwanted behaviour, confrontation, discriminating, refusal, denial, and threatening). Help-seeking is a strategy that includes informal social support seeking, informal organisational support seeking, and formal organisational support seeking. Informal social support seeking might include talking with friends, /someone for advice/support, and talking with family for understanding/support. Informal organisational support seeking includes talking with the supervisor/someone in management and reporting the situation informally. Finally, proper organisational support seeking is making a formal complaint. Finally, detachment, in this case, refers to leaving the situation or the perpetrators. It includes job mobility, keeping distance, and job and work withdrawal. On the other hand, barriers could be factors/conditions that limit women from exercising their coping mechanisms or the challenges they would face due to exercising these coping mechanisms (Fig. 1).

### Normalisation

Normalisation (avoiding or minimising conflict) is the process of considering workplace sexual harassment to a normal condition or state, which could be explained by accepting the workplace sexual harassment stands and agreement to invitation or offer. The elements are the act of willingly accepting the request, willingness to believe that the promise they have been told was real, and coming to terms with things that can come after sexual harassment. It could also include accepting workplace sexual harassment without protest, willingness to treat the perpetrator as a member of a group or social circle, and a positive response to the application. The findings show that normalisation has been the most frequently discussed coping dimension in sexual harassment. It contains several response elements: acceptance, ignorance, silence, negotiation, tolerance, and sorrow.

Many participants stated that employees’ acceptance of WSH was expected. The reasons were a lack of preventative strategies by management and the absence of sanctions for guests who harassed them. Many participants also reported fear of retribution, future revenge, and job loss in the perpetration acts by customers, agents, co-workers, and immediate bosses (supervisors, managers, owners). They also considered the normalisation elements of coping responses as critical job-related skills. They also believed that acceptance and ignorance of comments about physical attributes, tolerating touches and insults, accepting gestural signals, and crying in the case of hitting/slapping/pinching plays essential roles in coping with WSH:

[…], I considered workplace sexual harassment as a norm, ignore and keep silent for some of the activities of sexual harassment. Additionally, I accept perpetrators’ requests, appoint the perpetrators somewhere outside our workplace, willing to take phone numbers, and promise to continue without our interest. (FGD, four years’ experience in a cafeteria)

Since the customers are considered as kings and perceived that they are always right, mostly I keep silent for some acts of sexual harassment in my workplace (FGD, three years experience in a cafeteria)

Another participant added:

We are responsible for any inconveniences whoever made it. We would not be accepted for complaints because customers are income. Therefore, we did not challenge them. By any means, we have to make the situation calm. Because customers have absolute power. (FGD, two years of experience in a restaurant)

However, some participants mentioned that considering silence as a response was a challenge. They reported that some customers interpreted the silence as an agreement or acceptance of their requests for sexual harassment:

I do keep silent for most of the sexual harassment acts. I also know that some female employees stay quiet about sexual harassment acts. Nevertheless, being silent for customers/perpetrators is considered an acceptance of the request. (IDI, one and half years experience in a hotel)

Conversely, some participants mentioned that some factors helped them stick in this coping (Fig. 1). These were: promised to be promoted to a better job position, getting good recommendations, self-confidence and high morale, religious beliefs, and positive thinking and optimism:

Sometimes, managers will ask for sex. If we accept their request, they will write a good recommendation letter and let us go for the job in other hotels. (FGD, three years experience in a cafeteria)

### Engagement

This coping dimension refers to when the participants challenge the perpetrators face-to-face, especially in workplace sexual harassment, usually with hostility, criticism, or defiance. Victims confronted the harasser and told them to stop, or they are not interested. The analysis showed that engagement was found one of the crucial dimensions to cope with, recover from, and adapt to the effects of sexual harassment. This dimension encompasses a broad scope of sexual harassment coping elements such as accusing, confronting, discriminating, denying, refusing, and threatening the perpetrators. The respondents mentioned pinching the breasts and slapping the hips, insulting, requesting dates and sexual intercourse, and forceful kissing are typical sexual harassment in hospitality workplaces. Thus, they thought that the elements under this dimension were crucial:

I confront the perpetrators, discuss, and convince them that what they are doing is wrong and unacceptable. I also threaten them that I would expose them to the community. I would tell them that I know them before and that they have wives and children. These acts make them ashamed. This action would protect my friends and me in my institution. (IDI, 1-year experience in a restaurant)

However, the FGDs and IDIs participants mentioned the barriers to applying these coping dimensions, such as distributing nasty rumours about them. In addition, they were fired from the job, kicked, raped, slapped, insulted, and reduced their income due to practising this dimension’s coping elements.

Mostly, if we refuse to accept the perpetrator’s request through denial, confrontation, or discrimination, they would not come to our institution. It will mostly reduce the organisation’s income and might create bad rumours about the women employees in the organisation. (IDI, two years experience in the cafeteria)

### Help-seeking

This coping dimension revolves around determining women’s adaptation and coping strategies, which need third party involvement. This dimension includes informal social support seeking, informal organisational support seeking, and formal organisational support seeking. Informal social support seeking includes talking with friends/someone for advice/support and talking with family for understanding/support. Informal organisational support seeking includes talking with the supervisor/someone in management and reporting the situation informally. In conclusion, formal organisational support seeking is making a formal charge. The other component of this proportion was the religious aspect, such as praying. Help-seeking behaviour is a problem-focused, planned behaviour involving interpersonal interaction with selected stakeholders. Thus, victims first identify a problem that she perceives as requiring assistance and then seeks help. Finally, victims formally sought help by reporting the incident to the manager. Help-seeking behaviour has been defined as “a problem-focused, planned behaviour, involving interpersonal interaction with a selected healthcare professional.” For example, participants mentioned the following:

If the perpetrators (customers) action repeatedly occurs, I mostly inform my immediate supervisor or the manager. (IDI, 1-year experience in a restaurant)

I will report to my organisation to see if they can solve the problem. […]. If I were left out with no option in my organisation, I would report to the police. Most police are not responding to any sexual harassment activities, which happen in Infront of them. Also, sometimes the polices are the perpetrators on the road. So, the other solution that I left with is praying. (FGD, three years experience in a bar)

Similarly, participants mentioned that they talk with co-workers or intimate friends about a perpetrator, usually to exchange ideas or reach a conclusion, or talk of what they should do to a person. Victims mentioned that this communication was for support and advice:

Mostly, I communicate and discuss with my friend about the issue, and she supported me emotionally. She also convinces me that this job is temporary and all things would pass through time. It is not common to file a complaint to the legal body. (IDI, one year experience in a cafeteria)

### Detachments

In this dimension of coping, participants stay far enough away from perpetrators to be safe; to avoid becoming too friendly with customers/co-workers/supervisors/owners or brokers; disengage from their organisation when they become physically and psychologically, such as absenteeism, lateness/tardiness, and turnover; and move to different geographic locations for work, or change the work at all. Many FGD and IDI participants and key informants explained that the inactivity of the aforementioned coping strategies, bureaucracy, lack of complaints Procedure in organisations, managers’ influence, unfair treatment, and legal personnel’s perception were the situations in this patriarchal community. In reaction, women gave the operating area and place, withdraw from their task, and hold on to their distance from the culprits.

[…] Our last resort is to leave the job and compete in another place. (FGD, two years experience in a bar)

Most of the time, it is dissatisfying, inhibits our moral of the work. So, we will leave the job, and sometimes we will change the place of work until we experience a similar situation. (FGD, two years experience in a restaurant)

### Suggestions

There was a general agreement that these coping strategies were insufficient to cope with sexual harassment in hospitality workplaces. In addition, the lack of knowledge about sexual harassment, lack of complaint procedures, lack of strategies and policies, insufficient managerial skill, women who practice transactional sex, unfair treatment of women based on gender, victim-blaming, financial problems, and social discrimination were challenges.

The legislative institutions require witnesses from individuals who need t file a complaint, and there is unlikely to be any resolution for behaviours such as caressing, winking, or fondling. Those that observe this behaviour are afraid of losing their jobs f they do not witness it. Nevertheless, even if there is a witness, we are not ready to file a complaint. This non-accusation is due to our perception of the complaint’s lengthy process. We assumed that the legal process takes a long time and cost money. The legal authorities will take action if they see someone is hitting us. I think there is no legal issue with sexual harassment. Even if there is a legal process, it is not giving solutions. Some of the co-workers act like relationship creators. The other issue is that there are waitresses who are acting as commercial sex workers. (IDI, 1-year experience in a restaurant)

To surmount these roadblocks in coping dimensions, many IDI and FGD participants suggested various interventions based on the source of the barriers, such as organisational, stakeholder, government, and victim barriers. Since most of the problems are linked to hospitality workplaces, almost all participants indicated that organisations should adjust work shifts for female workers, establish formal complaint processes and training systems, formulate disciplinary procedures, improve employees’ wearing style and wages, and establish regular psychological counselling for employees. They also suggested posting awareness creation posters, respecting employee rights, and improving employee management.

I think awareness creation of sexual harassment before joining the work, formulation, and implementation of rules and regulations as well as complaint procedures, as well as posters to aware customers about sexual harassment and its impacts, can play significant roles. (IDI, 1-year experience in a cafeteria)

Another participant added:

One solution may be correcting all factors that push the customer towards sexual violence like the uniform-wearing style. Besides, there should be clear regulations in the hotels. (FGD, two years of experience in a restaurant)

Several participants also suggested that other stakeholders monitor actively and regularly, work on integration, dedicate promotional calendar, change the community’s attitude, empower women employees, and consider WSH prevention standards in their hospitality workplace levelling criteria.

I believe these organisations should be audited what they are doing in places such as restaurants, hotels, and other hospitality industries. (FGD, four years experience in a restaurant)

No, there is no. Nevertheless, we will be happy if we exist. We request stakeholders to control the salary scale and the influences of managers and business owners on waitresses. We are neglected groups. No one asks about our working conditions and the impacts we faced. (FGD, two years of experience in a cafeteria)

Another participant added:

As far as I know, waitresses are perceived to be wrong and responsible for all negative results. Whatever the case, customers are always right, which is illegal. There should be regulations that state the rights and responsibilities of the waitresses. We know our responsibility and working bit accordingly; we do not know our rights. Owners are worried about the duties of the waitresses, but not on our right. For every negative consequence, the responsible body should be identified. (FGD, three years experience in a grocery)

Furthermore, they suggest that the victims struggle for their rights by establishing an organisation that could fight for their rights and consults legal bodies, psychologists, and health care professionals while facing serious WSH.

I did not see any in my organisation. I think the solution is protecting ourselves. Even the police will not respond to any sexual harassment activities, which happen in Infront of them. Additionally, the polices are the perpetrators on the road. The other solution is praying. (FGD, five years experience in a restaurant)

Figure 1 shows the coping strategies and perceived barriers that emerged from the analysed qualitative data. The coping strategy contains four strategic dimensions for these specific women hospitality workers. These coping strategic dimensions are organised in a step-by-step approach considering the severity of the sexual harassment, the involvement of victims, perpetrators, hospitality workplaces, other organisations, and people. Moreover, the qualitative facts influenced the levelling of the dimensions and the links between them. Hence, normalisation and engagement dimensions were managed by either the individual employees (potential victims) or the perpetrators and applied for the minimum to moderate sexual harassment acts. In contrast, help-seeking and detachment dimensions were managed by the victims, the perpetrators, the organisations, and other stakeholders’ involvement and applied for relatively severe forms of sexual harassment. This coping strategy was developed through the interactive process among investigators who deeply immersed themselves in the data set and were subjected to critics and continuous feedback from experts, stakeholders, and representatives of the study communities.

## Discussion

This qualitative study explores coping strategies and perceived barriers to sexual harassment among women employees of Bahir Dar city hospitality workplaces, Ethiopia. To establish a productive coping strategy for WSH, understanding how women hospitality employees cope with sexual harassment is crucial. It is also vital to understand what barriers deter or facilitate their coping strategies. Coping WSH and barrier elimination attempts require context-driven strategies articulated through an in-depth understanding of local views and indigenous responses to WSH practices and respective barriers. Generally, the determinants of coping with WSH and the barriers are all local, with specific characteristics that must be realised for appropriate standards to reduce the risk of WSH [45]. However, most WSH prevention program interventions are guided by a broader global, regional, or national level framework [12], which lacks essential elements of sensitivity and compatibility with the target community’s local needs, priorities, and aspirations [67]. Such a top-down approach often fails to recognise the vital role of communities. It ignores the potential of local knowledge, resources, and capacities. They may saliently increase women’s employees’ vulnerability to multidimensional impacts of sexual harassment [3, 12, 68].

Given that the determinants, characteristics of vulnerability factors, and barriers vary based on occupation, gender, and cognition of the victims [45, 46], WSH reduction interventions that drive from contextually irrelevant strategies are fundamentally ineffective [17, 33, 45]. This study argues that the interventions targeted at reducing WSH and its impacts shall be supported by scheming and applying advanced strategies identified by scientific, research-oriented, and evidence-based coping strategies. This study inductively constructed coping strategies and barriers that can address the gaps in the science of WSH coping strategies, which are often guided by a limited understanding of the nature of the perpetrators, the causes of sexual harassment, the challenges of coping, and the socioeconomic characteristics of victims in specific settings. Thus, this study’s findings and the new framework are helpful to design and apply circumstantial and locally appropriate coping strategies to increase women’s employees’ capacity to withstand the consequences of WSH.

The current study results consist of four dimensions (normalisation, engagement, help-seeking, and detachment) of coping with WSH with perceived barriers and facilitators in each dimension application. The strategy shows the relationship among these coping dimensions in the context of hospitality WSH. Thus, it accentuates understanding what makes women employees unable to adapt to the specific dimensions of coping, making them less vulnerable to future risks and vulnerability and creating a specific opportunity for future interventions.

In this strategy, normalisation (avoiding or minimising conflict) has been identified as one of the most important coping strategies and constitutes coping elements of WSH and perceived barriers. Women employees come to see their experiences as usual and compare their experiences favourably with others. Consequently, they accept, keep silent, ignore, avoid, deny, tolerate, or sorrow while facing WSH in their workplace. There are perceptions that women’s employees’ tolerance and silence indicate interest and are facilitated by rewards such as promotion, helpful recommendation, self-confidence, religious beliefs, strong morals, positive networks, positive thinking, and optimism. Evidence also supports the idea that normalisation is preferred. This preference is due to the perception of hospitality employees’ acceptance of WSH as expected, considering the normalisation elements of coping responses as critical job-related skills, lack of preventative strategies by management, and an absence of sanctions for guests who harassed them [69]. However, these normalisation strategy dimensions were applied to all types of perpetrators-more for the managers, supervisors, owners, and co-workers. In line with the passive coping strategy [70], victims gently refused the perpetrator’s behaviour. Therefore, normalisation is the preferred dimension of coping for most victims. It contradicted the recommendation that suggests a firm and negative stance for the perpetrators [71].

Nevertheless, in hospitality workplaces, customers, or other perpetrators (co-workers and supervisors), the confrontation was considered wrong. Women may experience negative consequences of WSH due to their firm responses [70]. Additionally, sexual harassment, even in its severe form, was ignored and normalised at this stage. In line with other studies [16, 17, 33, 72], the possible reason was power differentiation. This finding implies that some of the victims of sexual harassment were complicit in an attempt to cover up the issue. The reason for not becoming bad was considering normalisation as the norm and one dimension of coping. In this regard, tailored interventions such as psychosocial interventions that buffer any negative mental health consequences of WSH, women’s empowerment, and awareness creation could build the employees’ capacity to choose the appropriate coping strategies. In fact, given the existence of deep-rooted and general beliefs and norms of WSH, the challenges to intervene at this stage are tough. The impact of such response elements on allying victims’ psychological problems remains unknown [73]. This challenge calls for active awareness creation movements about sexual harassment. Furthermore, future studies should investigate the effect of such responses in relieving victims’ psychological problems.

Engagement is among the essential coping strategy elements that appeared from the present data. Engagement consists of response components such as confronting, refusing, discriminating, threatening (blackmailing) or negotiating the perpetrators. It is the least used dimension. The potential reasons for its most minor usage were the barriers and consequences of engaging in this coping strategy dimension. The barriers were lack of knowledge on some legal backgrounds, lack of facilities to help in stressful situations, and surroundings’ inaccessibility.

The evidence also showed that women who confronted their harasser would be evaluated negatively by men and would be ascribed to more instrumental traits than women who did not confront the harasser, irrespective of WSH [17]. Women who confronted perpetrators were also considered lacking femininity and perceived as impertinent [17, 74]. Evidence also supports that retaliation, threatening the perpetrator in this study, is one of the coping methods of sexual harassment [75]. On the other hand, the feared consequences of applying this coping dimension were: the distribution of rumours, loss of a job, rape, physical harm, false accusation, income reduction/demotion, and verbal insults. These findings imply that women’s beliefs about the negative consequences and reactions deter many women from confronting the harasser and reporting the incident [17, 74]. Thus, ensuring legal mitigation measures and awareness about the formal ways of complaining of sexual harassment victimisation would minimise the women’s risks. Moreover, ensuring context-specific solidarity techniques would increase woman’s employees’ confidence and workplace communication with supervisors [75].

In the emerged coping strategy, help-seeking was the third dimension and appeared as a cornerstone. It contains essential response elements such as informal social support-seeking, informal organisational support seeking, and formal organisational and legal support-seeking. However, those who practised and were interested in practising this dimension of coping strategy faced challenges. These challenges were the wrong perception of legal bodies, deep-rooted beliefs, organisational norms, insufficient managerial skill, lack of complaint procedures, lack of financial resources, and societal acceptance/support. Evidence supports that the factors that most differentiated help-seeking dimension users from others were judgment and extent of the condition (i.e., Help-seekers found it to be far more frustrating, offensive, disturbing, and so forth, and the situation had persisted for weeks to months) [76]. Consistent with a study conducted in Australia [77], despite explicit legal frameworks for preventing and responding to WSH, women employees preferred extra-legal help-seeking practices in this study. As stated, the potential reasons for the preference for extra-legal help-seeking were bureaucracy-complex and time taking rules and regulations applied rigidly, the power difference between the victim and the perpetrator, corruption, fear of job loss, judicial reluctance and perception, ineffective legal assistance, and high cost. This finding implies that in addition to the low awareness about WSH’s legality, the implementation procedure also deters women from formal legal help-seeking behaviour. Thus, interventions that include awareness creation of legal bodies would help to increase legal help-seekers. However, this finding’s justification was beyond the limit of this research. Future research would see the challenges and solutions of the legal response to sexual harassment.

The fourth and final dimension of coping strategy that emerged in this study was detachment. This dimension consists of job-hopping, job withdrawal, work withdrawal, and distancing from the perpetrators. Those who practised and planned to practice this dimension faced challenges such as being hunted by the perpetrators, lack of employment opportunities, engaged in commercial sex work, and lack of financial resources. This dimension of coping with WSH was less addressed. In this study, the detachment was mentioned as the most frequent coping dimension. Consistent with the study on workplace incivility [76], this study’s detachment dimension practitioner shared specific characteristics with the practitioners’ minimisation dimension. However, unlike the mentioned study, the detachment dimension practitioners did not consider the WSH as mild. Instead, the response was for the more powerful perpetrators. The finding of this coping dimension implies that unemployment and other unemployment-related risks such as engaging in commercial sex work could increase. Thus, more organisations should provide counsellors with employee assistance programs targeted to employees, containing the emotional and occupational sequelae of workplace victimisation [51].

Also, this study shows that even silence is one of the coping strategies. It also explained that participants could use more than one dimension and component simultaneously or step-by-step. Furthermore, the barriers were significant for the shift from one dimension of coping with the other. Generally, this strategic framework could help in the development of context-specific WSH interventions. Nevertheless, future research should confirm the applicability of the framework using empirical studies.

However, this study has potential limitations. As the study participants were women who worked in hospitality workplaces, the views of men and other workplace contexts were not included, limiting the new conceptual framework’s adaptability. The framework is developed based on the perceptions and perspectives of women employees, supervisors, cashiers, and customers of hospitality workplaces and readers bear this in mind while interpreting the findings. Coping strategies should be shaped by the targets’ perspectives, realities, and priorities, ensuring receptivity and acceptability of the proposed strategic interventions. The strategic framework did not differentially treat people with disabilities; instead, it suggests interventions at the individual, organisation, and community levels, which could generally benefit hospitality workplace women employees. Moreover, the study did not cover large geographical areas, limiting the strategic framework’s scope of application. Another limitation of the study is that it focuses on women changing their behaviour and not men.

Furthermore, despite steps taken to assure participants felt safe and comfortable sharing information with researchers, some participants were still reluctant to reveal their experience. The team realised that this happened because the first author who led the women’s researchers’ team was a man. He led the team to ask women about workplace sexual harassment experiences, mainly stemmed from male customers, which needed to create an environment where women felt comfortable to share their authentic experiences of harassment. Therefore, to maintain the privacy, anonymity, and confidentiality of data, the first author explained to both team members and respondents that their identity and the evidence provided would be kept private. It was also made plain to the participants by the first author and team members that only the researchers directly involved with this study would have access to it. Then, they freely discussed the issues without fear of such conversations being monitored by their supervisors.

## Conclusions

Multiple barriers have challenged the response of women employees to WSH. The written report described that coping is multidimensional, having four interrelated dimensions with complex interactions and relationships, indicating that women employees may be enhanced by playing at different response pathways as possible entry levels for interference. The analysis indicated that coping does not live in a single category; instead, the entire framework, interacting with one another, constitutes coping. The framework is context specific, and it is advantageous to guide WSH response development to enable women employees to cope up, adjust to, and recover from the impacts. Building coping strategies against WSH requires interventions that strengthen the four components in concert and at multiple levels (individual, hospitality workplaces, and legal bodies). All coping strategic dimensions would cause a strong influence on both women employees and hospitality workplaces. However, strategic dimensions, such as normalisation and engagement, would substantially influence women employees. This framework would also help implement interventions that could eliminate the link between WSH and reproductive health impacts, such as the women’s employees’ involvement in transactional sex practice, commercial sex work, and prevent women from acquiring menstrual abnormalities and STIs. Therefore, any intervention needs to debate the barriers, facilitators, and outcomes related to response elements. While the framework offers a suitable direction to understand, lead up and design context specific response planning, it is likewise essential to test the applicability, conceptual and statistical relationship among the coping strategic dimensions through appropriate sample size and study method.

## Data Availability

The datasets used and/or analysed during the current study available from the corresponding author on reasonable request.

## Abbreviations

CIRHT: Center for International Reproductive Health Training
CSW: Commercial Sex Worker
FGDs: Focus Group Discussions
KIIs: Key informant Interviews
PhD: Doctor of Philosophy
PTSD: Post Traumatic Stress Disorder
STI: Sexually Transmitted Infections
WSH: Workplace Sexual Harassment
